# Multimodal Rehabilitation of Extensive Post‐Burn Scarring: A Case Report of Sequential Treatment With IPL, Microneedling + PRP, Ablative Fractional CO_2_ and Non‐Ablative Er,Yb:Glass

**DOI:** 10.1111/jocd.71172

**Published:** 2026-09-02

**Authors:** Carina Nogueira Garcia, Corinna Herz, Lidia Poppe

**Affiliations:** ^1^ Independent Researcher; ^2^ Hautärzte Am Königsplatz Augsburg Germany; ^3^ Dr. Poppe & Kollegen Dermatologie Praxis Bad Kissingen Germany

**Keywords:** burn scar, case report, combination therapy, fractional CO_2_ laser, hypertrophic scar, intense pulsed light, laser‐assisted drug delivery, microneedling, platelet‐rich plasma

## 1 Introduction

Hypertrophic scarring develops in up to 70% of survivors of significant burn injury and remains the greatest unmet challenge in burn care [1]. Deep burns rarely produce a uniform scar: a single territory typically contains hypertrophic ridging, atrophic depression, telangiectasia and dyspigmentation, reflecting regional variation in burn depth and inflammatory load [2]. Among 1034 people living with scars, the 119 with burn scarring reported more symptoms and greater psychological difficulty than those with other etiologies [3].

No single modality addresses this heterogeneity, so multimodal protocols have become the pragmatic standard. What remains undefined is sequencing. A meta‐analysis of seven studies and 467 patients found that laser therapy begun within 12 months of injury outperformed later intervention (standardized mean difference −1.97, 95% CI −3.08 to −0.87, vs. −0.59, −1.10 to −0.07), with vascularity the most timing‐sensitive subscale [4]. Timing and order are therefore determinants of outcome, not administrative detail.

The available modalities act on complementary targets. Intense pulsed light (IPL) permits selective photothermolysis of oxyhemoglobin and melanin, with reported use in hypertrophic scarring and post‐inflammatory dyschromia [5]. In a cohort of 20 patients with burn scar dyschromia, 16 improved [6]. Because hypervascularity sustains fibroproliferation, vascular and collagen targets are mechanistically linked [7].

Ablative fractional CO_2_ laser (AFCL) ablates microcolumns while preserving tissue bridges. In mature burn scars it improved collagen architecture and shifted the Type I:III ratio [8], and pooled data across 14 studies show a mean Vancouver Scar Scale (VSS) reduction of 3.01 points (95% CI −3.79 to −2.22) [9]. Early intervention in acute burns produced 63% improvement on the Manchester Scar Scale [10].

Evidence for combining light devices is weaker than assumed: a 2025 review admitted only two studies to quantitative synthesis and found no advantage of combination over pulsed dye laser alone [11]. Hybrid platforms delivering ablative CO_2_ and non‐ablative 1570 nm Erbium glass from one device are a newer option. In 51 patients this pairing improved pigmentation, vascularity, and elasticity alongside itch, pain, and tension [12]. Platelet‐rich plasma (PRP) supplies PDGF, VEGF, TGF‐β, and IGF‐1 [13], and adding PRP to microneedling nearly tripled the odds of > 50% improvement across 14 controlled studies (OR 2.97; 95% CI 1.96–4.51) [14].

We describe a phased 10‐session protocol in which IPL and microneedling with PRP preceded AFCL, and in which each laser session served as a delivery window for same‐session mesotherapy. The report follows the CARE guidelines [15].

## 2 Case Report

### 2.1 Patient Information

An 18‐year‐old woman (Fitzpatrick Type II) presented 10 months after a motor vehicle accident caused deep partial‐thickness (second‐degree) burns (formerly graded as 2B) [2] over approximately 20% of the total body surface area, predominantly in the medial thighs. She had no history of keloid formation, dermatological or autoimmune disease, and took no systemic medication. Acute management comprised 6 weeks of enzymatic and surgical debridement. Grafting was not required, and closure by secondary intention was complete at 4 months. At baseline she had been treated with compression garments.

### 2.2 Clinical Findings

**FIGURE 1:**
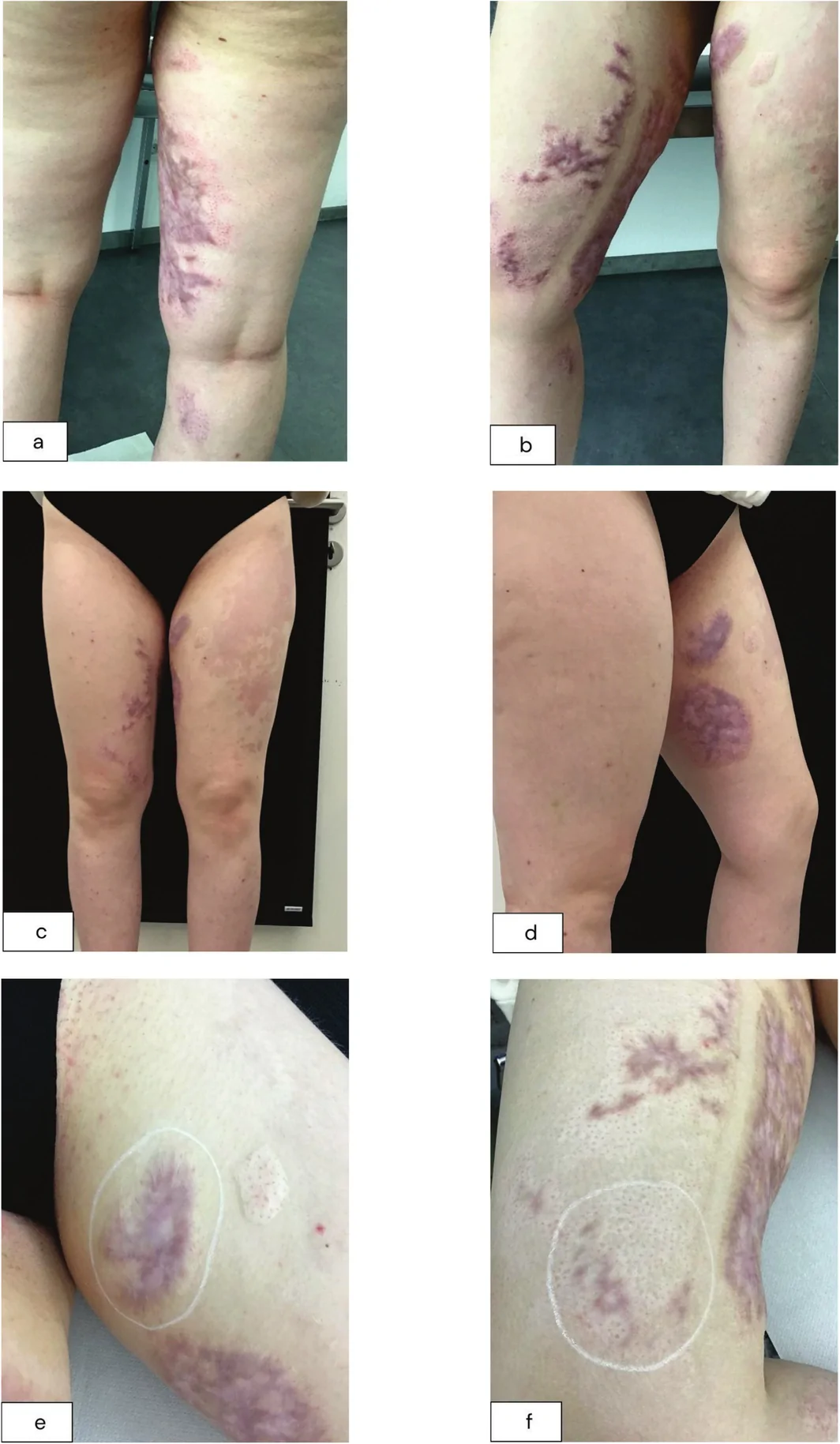
Baseline appearance of the bilateral thigh scar field at 10 months post‐injury in use of compression garments, showing coexisting hypertrophic plaques, atrophic depressions, diffuse telangiectasia and post‐inflammatory hyperpigmentation. (a) Right thigh posterior. (b) Right thigh medial (June 2021). (c) Naevus Flammeus (Port Wine Stain) seen on the anterior region of the left thigh. (d) Left thigh medial, two distinguished scar areas (October 2021). (e, f) Selected areas circled in white for the IPL trial session (October 2021).

Examination showed a Naevus Flammeus (Port Wine Stain) on the anterior region of the left thigh that the patient reported having since birth. The findings related to the complex burn scar were four coexisting components: firm hypertrophic plaques with a red‐to‐violaceous hue; atrophic depressions with thin, fragile epithelium; diffuse telangiectasia and erythema; and patchy post‐inflammatory hyperpigmentation. The patient reported burning discomfort on contact, pruritus, and marked psychosocial distress. The baseline VSS score [16] was 9/15 (vascularity 3, pigmentation 2, pliability 2, height 2) seen in Figure 1. One clinician performed all assessments, with standardized photography before every session.

### 2.3 Therapeutic Intervention

The treatment was performed from October 2021 until November 2025, in 10 sessions grouped in three phases (Table 1; parameters in Table 2). Phase 1 addressed vascular and pigmentation components with three IPL sessions. Phase 2 consisted of three microneedling and PRP sessions, the intent being to biologically prime the tissue before ablative energy. PRP was prepared from 15 mL of autologous blood by double centrifugation, yielding approximately 7 mL at four to six times baseline platelet concentration, and applied topically during and after microneedling at 1.5–2.0 mm. Phase 3 comprised four laser sessions: two of AFCL and two on a hybrid platform delivering ablative CO_2_ and non‐ablative 1570 nm Erbium glass in the same pass, chosen to pair residual surface remodeling with deeper non‐ablative dermal heating. Each Phase 3 session was immediately followed within the same visit by intradermal mesotherapy with botulinum toxin type A 4 IU, non‐crosslinked hyaluronic acid and an injectable polynucleotide. All sessions were followed by 15 min of cooling. Clobetasol propionate 0.05% was applied for 5 days after IPL and laser sessions but withheld after PRP so as not to blunt the intended healing response. Photoprotection was mandated throughout.

**TABLE 1:** Sequential treatment protocol: Session‐by‐session clinical workflow.

Session	Date	Phase	Modality	Primary target	Treated areas	Post‐procedural care	Observed response
1–Trial	October, 2021	1	IPL	Telangiectasia, erythema, PIH	Selected test areas (Figure 1e,f)	Cooling 15 min; clobetasol 0.05% BD × 5 days	Immediate purpura of ectatic vessels; erythema resolving by day 5
2	October, 2021	1	IPL	Telangiectasia, erythema, PIH	Anterior‐medial right thigh; medial left thigh	Cooling 15 min; clobetasol 0.05% BD × 5 days	Further reduction in vessel caliber and density
3	December, 2021	1	IPL	Residual telangiectasia and dyschromia	Posterior‐medial right thigh; medial left thigh	Cooling 15 min; clobetasol 0.05% BD × 5 days	Color shift red‐violaceous → pink; VSS vascularity 3 → 2
4	March, 2022	2	Microneedling + PRP	Global field: collagen induction, pliability	Medial thigh bilateral	Cooling; emollient only (no corticosteroid)	Pinpoint bleeding endpoint achieved; edema resolved < 72 h
5	May, 2022	2	Microneedling + PRP	Global field: collagen induction, pliability	Medial left thigh	Cooling; emollient only	Early softening of hypertrophic plaques on palpation
6	June, 2022	2	Microneedling + PRP	Global field; symptom control	Right leg	Cooling; emollient only	Contact burning discomfort markedly reduced
—	October, 2023	Interim	Assessment only (Figure 2)	VSS + standardized photography	Medial thigh bilateral	—	End of Phases 1 and 2—VSS 6/15 (V2 P1 Pl1 H2)
7	November, 2024	3	FRXLCO_2_ + mesotherapy	Hypertrophic contour; atrophic texture	Medial thigh bilateral	Cooling 15 min; clobetasol 0.05% BD × 5 days	Re‐epithelialization complete by day 5–7; no dyspigmentation
8	December, 2024	3	FRXLCO_2_ + mesotherapy	Hypertrophic contour; atrophic texture	Medial thigh bilateral	Cooling 15 min; clobetasol 0.05% BD × 5 days	Progressive flattening of hypertrophic plaques
9	October, 2025	3	Hybrid: ablative CO_2_ + non‐ablative 1570 nm Er,Yb:Glass + mesotherapy	Residual elevation and surface irregularity	Medial thigh bilateral	Cooling 15 min; clobetasol 0.05% BD × 5 days	Smoothing of atrophic depressions; pliability near normal
10	November,2025	3	Hybrid: ablative CO_2_ + non‐ablative 1570 nm Er,Yb:Glass + mesotherapy	Residual hypertrophic margins	Medial thigh bilateral; medial right lower leg	Cooling 15 min; clobetasol 0.05% BD × 5 days	Final session; no adverse events
—	December, 2025	Follow‐up	Assessment only (Figure 3)	VSS + standardized photography	—	Photoprotection SPF 50+ continued	Final VSS 3/15 (V1 P1 Pl0 H1); 67% reduction

*Note:* All sessions were performed with at least four‐week intervals. Mesotherapy was delivered intradermally immediately after each CO_2_ laser pass within the same session (botulinum toxin type A 4 IU, non‐crosslinked hyaluronic acid, polynucleotide injectable). Device parameters are detailed in Table 2.

Abbreviations: BD, twice daily; CO_2_, carbon dioxide; FRXL, fractional; H, height; IPL, intense pulsed light; P, pigmentation; PIH, post‐inflammatory hyperpigmentation; Pl, pliability; PRP, platelet‐rich plasma; V, vascularity; VSS, Vancouver Scar Scale.

**TABLE 2:** Device settings, preparation protocols and mechanistic rationale for each modality.

Modality	Parameters and preparation	Sessions	Mechanistic rationale
Intense pulsed light	Alma Rejuve; 515–590 nm cut‐off filter; fluence 6–7 J/cm^2^; pulse duration 12–15 ms; double‐pulse mode	3	Selective photothermolysis of oxyhemoglobin in ectatic vessels and of melanin in dyschromic tissue; reduction of the hypervascular drive to fibroproliferation
Microneedling with PRP	Dermaroller, needle depth 1.5–2.0 mm, stamping pattern to uniform pinpoint bleeding; PRP from 15 mL autologous blood by double centrifugation (1500 rpm × 5 min, then 2500 rpm × 10 min) → ~7 mL at 4–6× baseline platelet concentration, applied topically during and after microneedling	3	Percutaneous collagen induction plus transdermal delivery of PDGF, VEGF, TGF‐β and IGF‐1 through the micro‐channels
Ablative fractional CO_2_ laser	Exelo, Alma Hybrid; 10 600 nm; 20 W; 30 J/cm^2^ per microbeam; pulse duration 2 ms; frequency 1 kHz; density 150 points/cm^2^, regionally adjusted (higher density over hypertrophic zones, superficial pass over atrophic zones)	2	Pixelated microcolumn ablation with intervening tissue bridges; collagen reorganization and normalization of the Type I:III ratio
Combination ablative CO_2_ and non‐ablative Erbium‐Glas laser	Alma Hybrid: AFCL CO_2_ (10 600 nm, 20 W; 30 J/cm^2^ per microbeam; pulse duration 2 ms; frequency 1 kHz; density 150 points/cm^2^) + Er,Yb:Glass (1570 nm 10 W; 4 ms on‐time; 40 mJ). Ultrasound IMPACT technology (50 Hz; 2 min) applied after drug delivery.	2	Superficial ablative resurfacing paired with deeper non‐ablative dermal heating at lower epidermal cost; used once bulk hypertrophy had resolved and the residual deficit was textural
Same‐session mesotherapy	Delivered intradermally immediately after each CO_2_ pass: botulinum toxin type A 4 IU (Botox) per session as multiple microaliquots; non‐crosslinked hyaluronic acid (MesoHA); polynucleotide‐based injectable (PhilArt)	4	Laser‐assisted drug delivery through transient microchannels; botulinum toxin for mechanical tension and fibroblast modulation; hyaluronic acid for hydration and matrix support; polynucleotides for fibroblast stimulation
Adjunctive care	Chilled saline compresses and medical‐grade cold air, 15 min after every session; clobetasol propionate 0.05% cream BD × 5 days after IPL and CO_2_ sessions only; broad‐spectrum SPF 50+ and physical photoprotection throughout	All 10 sessions	Attenuation of the post‐procedural inflammatory peak and of post‐inflammatory hyperpigmentation risk; corticosteroid withheld after PRP sessions to preserve the intended healing response

Abbreviations: BD, twice daily; IGF‐1, insulin‐like growth factor 1; IPL, intense pulsed light; PDGF, platelet‐derived growth factor; PRP, platelet‐rich plasma; SPF, sun protection factor; TGF‐β, transforming growth factor beta; VEGF, vascular endothelial growth factor.

### 2.4 Follow‐Up and Outcomes

**FIGURE 2:**
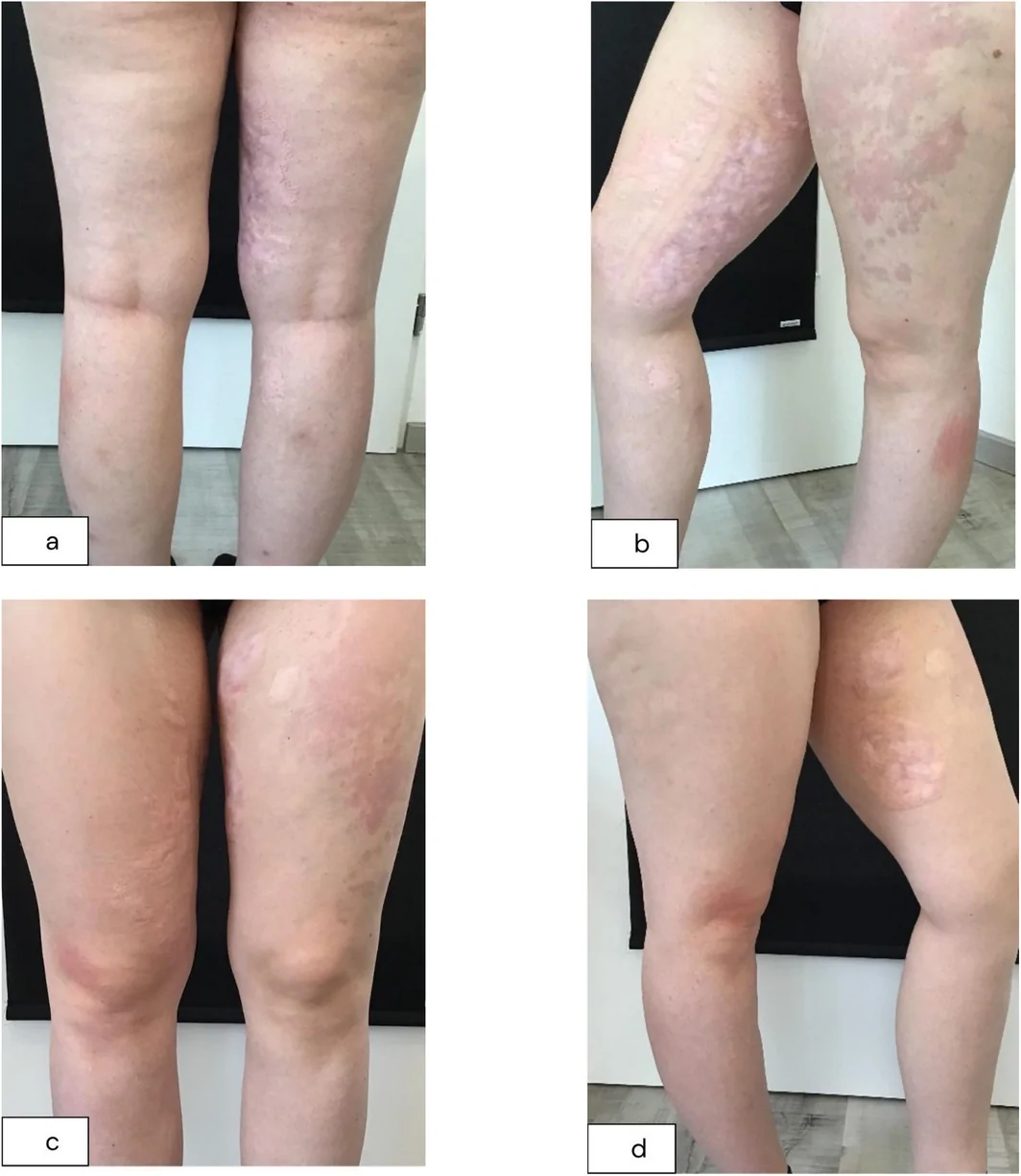
Interim assessment in October 2023 after end of Phases 1 (3 sessions of IPL) and 2 (3 sessions of microneedling + PRP) − VSS 6/15 (V2 P1 Pl1 H2) showing reduced erythema, telangiectatic density, and post‐inflammatory hyperpigmentation. (a) Legs posterior view, (b) right thigh medial, left thigh lateral, (c) thighs anterior view, (d) left thigh medial and right thigh lateral.

**FIGURE 3:**
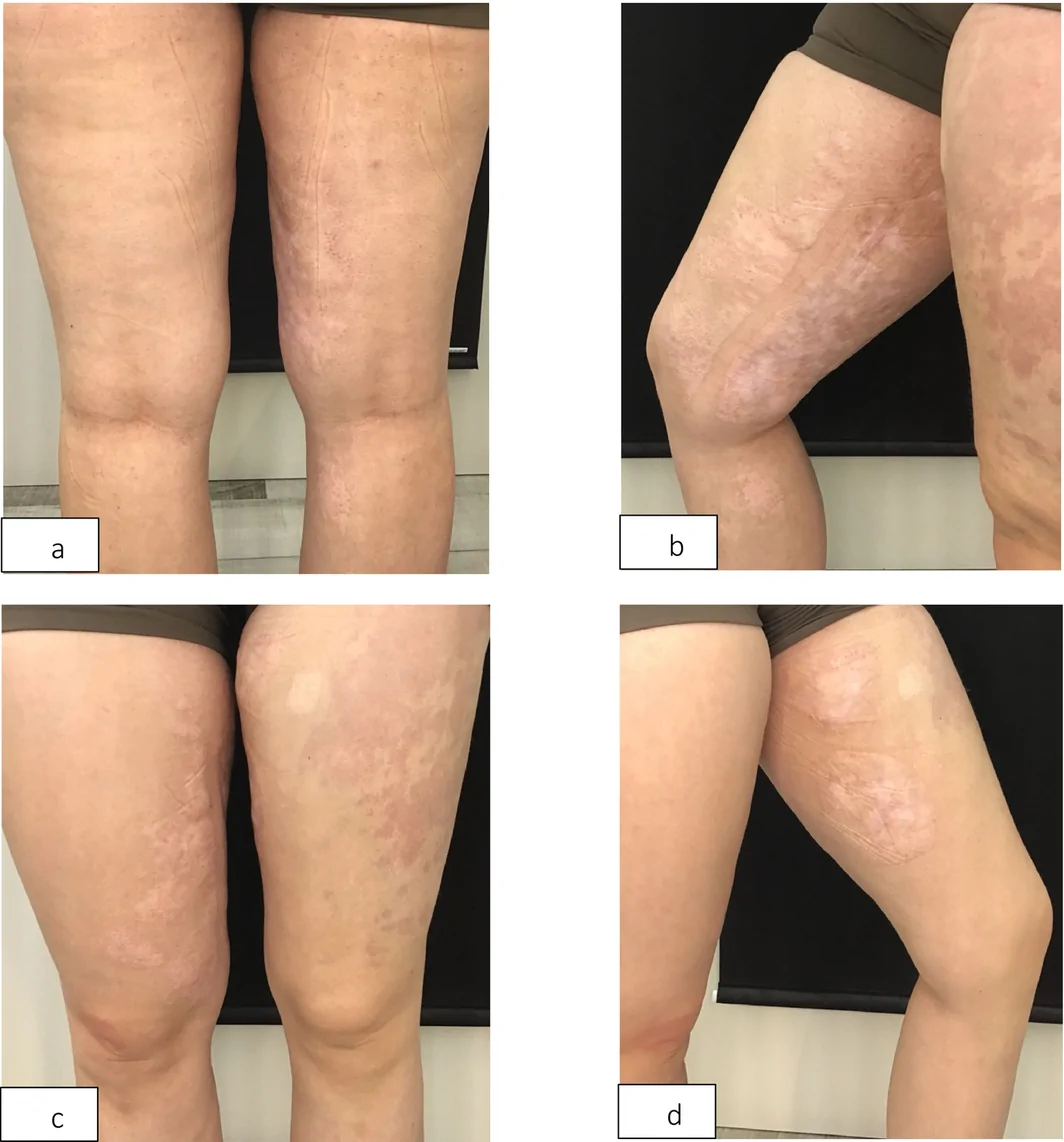
Final appearance after Phase 3 (2 AFCL sessions, 2 non‐ablative Erbium Glass sessions, same‐session mesotherapy) showing flattening of hypertrophic plaques and smoothing of atrophic zones. (a) Thighs posterior view, (b) right thigh medial, (c) thighs anterior view, (d) left thigh medial.

**FIGURE 4:**
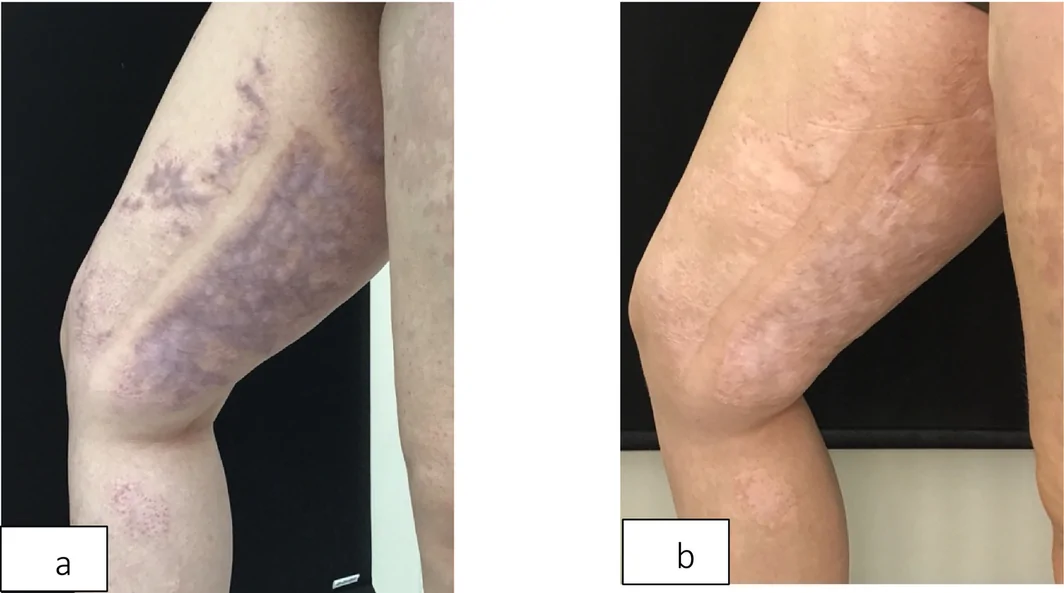
Zoomed‐in comparison of right thigh medial region showing clearing of violaceous appearance due to erythema and telangiectasia and flattening of hypertrophic plaques from baseline in (a) October 2021 to final assessment in (b) November 2025.

**FIGURE 5:**
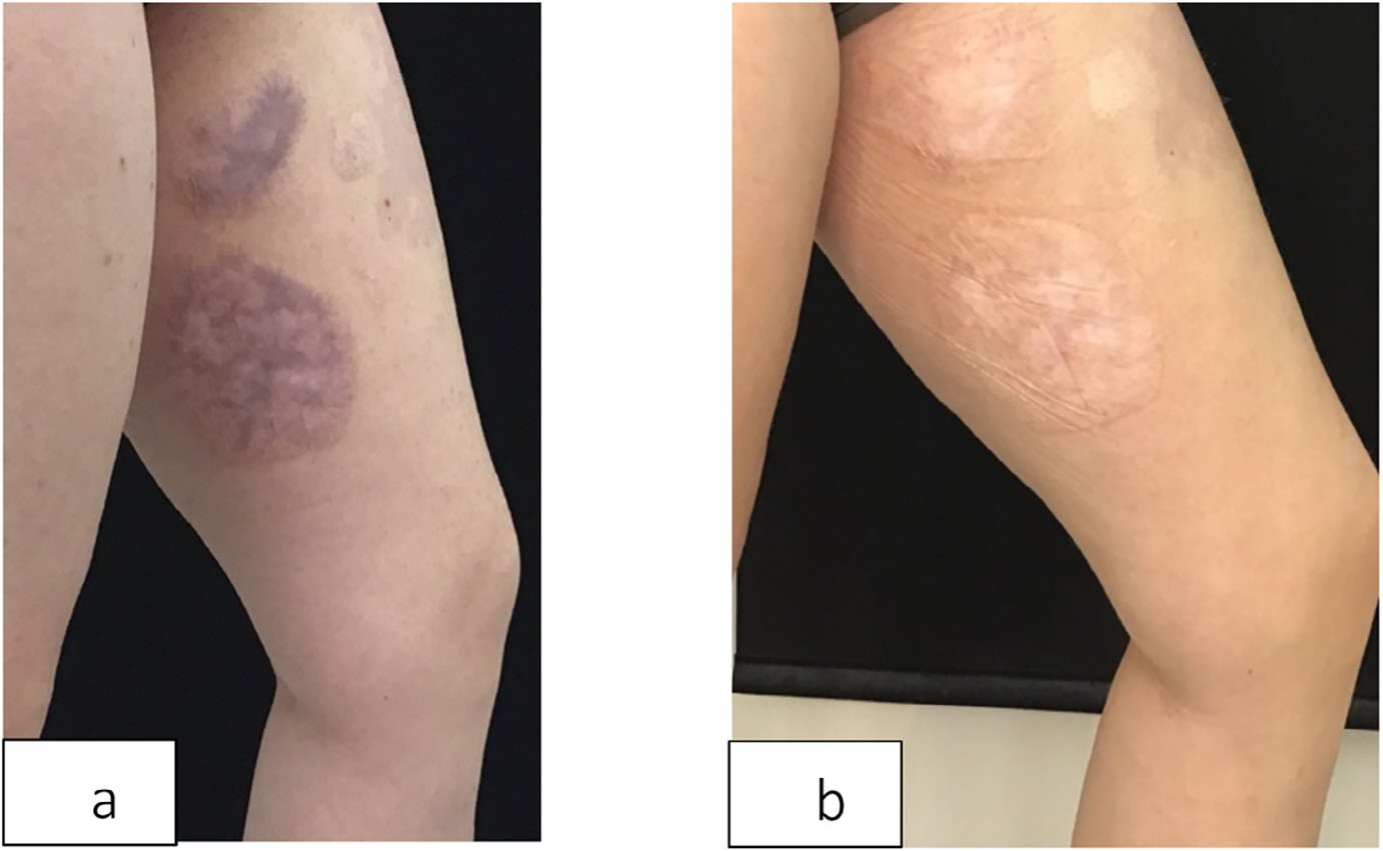
Zoomed‐in comparison of left thigh medial region showing clearing of violaceous appearance due to erythema and telangiectasia and flattening of hypertrophic plaques from baseline in (a) October 2021 to final assessment in (b) November 2025.

Erythema and telangiectasia responded earliest, with reduced vessel density and a color shift from red‐violaceous to pink by the end of Phase 1; pliability and contact discomfort improved during the same phase. Structural change followed Phase 2, with flattening of hypertrophic plaques and smoothing of atrophic depressions seen in Figure 2. The final VSS score was 3/15 (Figure 3), a 67% reduction, improving in every subscale (vascularity 3 → 1, pigmentation 2 → 1, pliability 2 → 0, height 2 → 1) as observed in Figures 4 and 5. The patient reported resolution of discomfort and substantial improvement in self‐image. Adverse events were limited to transient erythema and oedema resolving within 72 h. No blistering, infection, dyspigmentation or recurrence occurred. Histology was not obtained. The patient remains on follow‐up.

## 3 Discussion

The 67% VSS reduction observed here compares favorably with the pooled 29% improvement reported across 282 patients treated with AFCL alone [17], though an uncontrolled single case cannot establish superiority over monotherapy [15]. This report contributes with a transparent account of the sequencing process, which is presented in a tabulated form because order and spacing are precisely what published series most often omit.

Treating the vascular component first was intended to reduce inflammatory burden before ablation. Vascularity showed the earliest and largest movement, consistent both with IPL's mechanism [5] and with the meta‐analytic finding that this subscale is the most timing‐sensitive [4]. This ordering is mechanistically reasoned rather than evidence‐based: comparative data on light‐device sequencing in burn scars remain sparse [11]. Completing a phase of microneedling with PRP before ablation aimed to improve tissue quality in advance of thermal injury, exploiting the micro‐channels as both collagen‐induction stimulus and delivery route [18]. In a rat model of full‐thickness burn scarring, the combination outperformed either component alone in matrix organization and angiogenesis [19].

The structural gains followed AFCL, consistent with its histologically demonstrated remodeling of collagen architecture [8]. Concluding with a hybrid session was a deliberate de‐escalation: by that point the residual deficit was textural rather than bulk hypertrophy and combining CO_2_ with 1570 nm Erbium glass paired superficial resurfacing with deeper non‐ablative heating in one pass. In 51 patients treated with this pairing, improvement spanned pigmentation, vascularity, and elasticity as well as itch and tension [12]. Fractional laser also reduces melanin index measured spectrophotometrically across Fitzpatrick types [20], which is reassuring when hyperpigmentation is the dominant procedural risk.

Phototype II influenced parameter choice. The risk of post‐inflammatory hyperpigmentation is lower than in darker skin, permitting the settings used without staged test spotting. The trade‐off is a persistent erythema tendency and starker vascular contrast, which is why vascularity dominated the baseline VSS at 3 of 3 and why IPL led the protocol. Clobetasol and photoprotection were retained regardless, neither being dispensable in fair skin undergoing repeated ablation.

The distinctive element is same session mesotherapy. Laser‐assisted drug delivery exploits transient microchannels that close within hours, so simultaneous administration captures a pharmacokinetic advantage a separate visit cannot; a scoping review of 55 publications confirms the technique is now routine in hypertrophic scar management, albeit with heterogeneous agents and endpoints [21]. Each component has independent support: botulinum toxin type A improved VSS by a mean of 1.07 points across 19 studies and 686 patients [22], non‐crosslinked hyaluronic acid improved erythema and texture in a split‐face randomized trial [23], and polynucleotides are used for scar management by 73% of 501 surveyed dermatologists, though in hypertrophic scars by only 10% [24]. Administered together, their individual contributions cannot be separated; the cocktail is an empirical composite, not a validated formulation.

This protocol combines within a session where delivery depends on simultaneity but separates thermal from biological modalities into distinct phases so that burn‐damaged skin is not subjected to cumulative injury and response could be reassessed at each interval. The evidence does not settle this: a 2026 systematic review of AFCL with PRP excluded non‐synchronous administration altogether [25], and the meta‐analysis in atrophic acne scarring found significant benefit for synchronous combination (OR 2.56; 95% CI 1.37–4.78) [26]. Whether phase separation confers a safety advantage that offsets any loss of synergy in burn‐damaged skin is untested.

The psychosocial dimension deserves explicit weight. This was an adolescent with disfiguring scarring of a body region governing clothing choice and physical participation, and survey evidence indicates such burden persists after discharge from acute services [3]. Her reported gains in self‐image were, from her perspective, at least as important as the change in VSS, a dimension the scale does not capture.

### 3.1 Limitations

Several limitations apply. This is a single uncontrolled observation, so treatment effect cannot be separated from natural maturation over a real concern given that responsiveness itself varies with scar age [4]. Multiple modalities were combined, making attribution impossible by design. Assessment relied on one unblinded observer using an instrument with moderate inter‐rater reliability for scar assessment without considering patient‐reported dimension [27, 28]. No instrumental measurement or histology was obtained.

## 4 Conclusion

A phased protocol, three IPL sessions followed by three microneedling and PRP sessions, then two AFCL sessions concluding with two hybrid ablative and non‐ablative sessions, each with same‐session mesotherapy, was associated with a 67% VSS reduction and improvement in all four subscales, without adverse events beyond transient erythema and edema. Two elements may merit wider consideration: reducing vascular burden and biologically priming tissue before ablative energy and exploiting each ablated field as a same‐session delivery window. Both are hypotheses from a single case and require controlled evaluation.

## Author Contributions

**Carina Nogueira Garcia:** conceptualization, data curation, investigation, writing – original draft. **Corinna Herz:** clinical consultation, clinical management, and writing – review and editing. **Lidia Poppe:** clinical consultation, clinical management, conceptualization, writing – review and editing. All authors approved the final version of the manuscript and agreed to be accountable for all aspects of the work.

## Funding

The authors have nothing to report.

## Disclosure

Reporting Guideline: This report was prepared in accordance with the CARE guidelines for case reports.

## Ethics Statement

The authors confirm that the ethical policies of the *Journal of Cosmetic Dermatology* have been adhered to. According to institutional guidelines, formal ethics committee approval is not required for case reports involving one or two patients, as long as no identifiable personal data beyond clinical images are used. This study complies with the Declaration of Helsinki principles.

## Consent

Written informed consent was obtained from the patient for the publication of her clinical details and accompanying images.

## Conflicts of Interest

The authors declare no conflicts of interest.

## Data Availability

Data sharing is not applicable, as this article reports individual clinical case and no datasets were generated or analyzed.
